# Acute Kidney Injury in Adults Exposed to Commonly Used Nephrotoxic Medications and Iodinated Contrast: Frequency, Associated Factors, and Early Outcomes

**DOI:** 10.7759/cureus.111155

**Published:** 2026-06-19

**Authors:** Madala Varun, Saieesha Chowdary Kolla, Aalasyam Naveen, Prakhya Chowdary Koya, Suresh Babu Sayana

**Affiliations:** 1 Medicine, All India Institute of Medical Sciences, Mangalagiri, IND; 2 Medicine, Mamata Medical College, Khammam, IND; 3 Pharmacology, Mamata Medical College, Khammam, IND; 4 Medicine, University of Pittsburgh, Pittsburgh, USA; 5 Pharmacology, Government Medical College and General Hospital, Bhadradri Kothagudem, IND

**Keywords:** acute kidney injury, inpatient renal monitoring, iodinated contrast, nephrotoxic medications, nephrotoxin stewardship, propensity-score matching, vancomycin

## Abstract

Background

Acute kidney injury (AKI) in hospitalized adults is often framed as an unavoidable consequence of critical illness. Yet a substantial proportion emerges within a modifiable clinical space: exposure to nephrotoxic medications, iodinated contrast, hemodynamic stress, and delayed renal monitoring. This study evaluated whether high nephrotoxic exposure was associated with in-hospital AKI among adult inpatients after balancing measured baseline risk.

Methods

This prospective observational cohort study included adult inpatients admitted to general wards and intensive care units at Mamata Medical College and General Hospital, Khammam, India. From 1,500 admissions, 240 patients with high nephrotoxic medication exposure and/or iodinated contrast exposure were identified and matched 1:1 with 240 unexposed controls using propensity-score matching. Matching variables included age, sex, body mass index, diabetes mellitus, congestive heart failure, baseline serum creatinine, estimated glomerular filtration rate, blood urea nitrogen, and intensive care unit status. AKI was defined according to the Kidney Disease: Improving Global Outcomes criteria, primarily using serum creatinine changes. A sensitivity analysis excluded very mild Stage 1a AKI. The primary outcome was in-hospital AKI after the index date; secondary outcomes included AKI severity, Stages 2-3 AKI, time to AKI among AKI cases, in-hospital mortality, length of stay, vancomycin concentration patterns, and medication-specific AKI-overlapping exposure burden.

Results

After matching, baseline characteristics were generally balanced, although blood urea nitrogen remained modestly imbalanced. AKI occurred in 61 exposed patients and 38 controls, corresponding to 25.4% versus 15.8% and an odds ratio of 1.81 (95% CI, 1.15-2.85; p = 0.013). Severe AKI was numerically higher in exposed patients but did not reach statistical significance. After excluding Stage 1a AKI, clinically meaningful AKI remained more frequent in the exposed group, 17.5% versus 10.0% (OR, 1.91; 95% CI, 1.12-3.27; p = 0.024). Vancomycin, iodinated contrast, piperacillin-tazobactam, antivirals, and calcineurin/mammalian target of rapamycin (mTOR) inhibitors contributed prominently to AKI-overlapping exposure days. Maximum vancomycin concentrations were higher among patients who developed AKI, although reverse causality could not be excluded.

Conclusion

High nephrotoxic medication and/or iodinated contrast exposure was associated with a higher frequency of in-hospital AKI in matched adult inpatients. The association persisted after excluding minor creatinine-only events, supporting a clinically meaningful renal injury signal. These findings strengthen the need for nephrotoxin stewardship, early creatinine surveillance, dose adjustment, hydration optimization, and active multidisciplinary review when renal-risk medications are clinically unavoidable.

## Introduction

Acute kidney injury (AKI) remains one of the most preventable yet frequently missed complications of hospital care. It is not a single disease, but a clinical signal of reduced renal reserve, disturbed perfusion, inflammatory stress, and exposure to potentially nephrotoxic drugs or iodinated contrast. Global evidence has consistently shown that AKI increases mortality, length of stay, dialysis requirement, and later chronic kidney disease risk [1,2]. Importantly, a meaningful proportion of hospital-acquired AKI may be avoidable through timely volume assessment, correction of hemodynamic instability, contrast prophylaxis, renal-dose adjustment, and avoidance of unnecessary nephrotoxic combinations [3]. In routine wards and intensive care units, this risk becomes sharper because patients often receive antibiotics, renin-angiotensin system blockers, nonsteroidal anti-inflammatory drugs, antivirals, calcineurin/mammalian target of rapamycin (mTOR) inhibitors, vancomycin, piperacillin-tazobactam, and radiocontrast within a short clinical window [4-6]. Mechanistically, drug-induced AKI may occur through acute tubular injury, acute interstitial nephritis, crystal nephropathy, altered intraglomerular hemodynamics, and drug accumulation in vulnerable tubular cells [4,7,8].

The clinical problem is not only toxicity. It is recognition. Several hospital-based studies have shown that mild or biochemical AKI is often underdiagnosed, although it still carries prognostic weight [9-11]. Adult adaptation of nephrotoxin-surveillance approaches such as Nephrotoxic Injury Negated by Just-in-Time Action (NINJA) has further shown that high nephrotoxic exposure is associated with higher AKI frequency, including severe AKI and mortality signals, with vancomycin, iodinated contrast, and piperacillin-tazobactam contributing substantially to exposure burden [12]. However, causal interpretation remains difficult because nephrotoxin use is strongly linked with illness severity, sepsis, hemodynamic instability, comorbidity, and longer hospitalization. Therefore, the present study was designed to determine whether high nephrotoxic medication exposure and/or iodinated radiocontrast exposure were associated with increased in-hospital AKI among adult inpatients after propensity-score matching. The primary objective was to compare AKI frequency between exposed admissions and matched unexposed controls. Secondary objectives were to evaluate AKI severity, Stages 2-3 AKI, time to AKI among AKI cases, in-hospital mortality, length of stay, and vancomycin concentration patterns.

## Materials and methods

This prospective observational cohort study was conducted among adult inpatients admitted to the general wards and intensive care units of Mamata Medical College and General Hospital, Khammam, Telangana, India. The study was designed to evaluate the association between qualifying nephrotoxic medication or iodinated contrast exposure and subsequent AKI during hospitalization. Institutional Ethics Committee approval was obtained before study initiation (MMC/IEC/06/2026 dated 27 January 2026). As this was an observational study, patient management, drug selection, dosing, and radiological decisions were made by the treating clinical teams according to routine hospital practice.

Study population

Adult inpatients aged 18 years or older were screened for eligibility. Patients were included if they had a documented baseline serum creatinine before nephrotoxic exposure and either received at least one predefined nephrotoxic medication for 24 hours or more or received iodinated radiocontrast during hospitalization. The baseline serum creatinine was defined as the most recent serum creatinine value available before the qualifying nephrotoxic exposure or contrast administration.

Patients were excluded if they had end-stage kidney disease on maintenance dialysis, prior kidney transplantation, pregnancy, urinary tract obstruction, AKI already present before nephrotoxic exposure, or inadequate renal follow-up data after exposure. Admissions without sufficient serum creatinine monitoring to determine AKI status were also excluded. The pre-matching cohort included 1,500 adult inpatient admissions. Among these, 240 admissions met the criteria for high nephrotoxic exposure and were matched to 240 control admissions using propensity-score matching. Control admissions were defined as admissions without qualifying nephrotoxic medication exposure or iodinated contrast exposure before the assigned index date.

Nephrotoxic exposure assessment

Nephrotoxic exposure was defined a priori using commonly recognized inpatient nephrotoxic agents and iodinated radiocontrast. The medication classes included angiotensin-converting enzyme inhibitors or angiotensin receptor blockers, aminoglycosides, vancomycin, piperacillin-tazobactam, antiviral agents, calcineurin inhibitors, mTOR inhibitors, nonsteroidal anti-inflammatory drugs, iodinated contrast agents, and Other nephrotoxic medications were included only if they were prespecified in the study extraction sheet and were documented in the medication administration record.

For each eligible admission, the drug name, dose, route of administration, timing of initiation, duration of therapy, and concurrent nephrotoxic medication use were recorded where available. For patients exposed to iodinated contrast, the type of contrast agent and timing of administration were documented. High nephrotoxic exposure was defined as receipt of at least one prespecified nephrotoxic medication for 24 hours or more, or receipt of iodinated radiocontrast during hospitalization. For exposed admissions, the index date was defined as the date of first qualifying nephrotoxic medication exposure or iodinated contrast administration. For matched control admissions, a corresponding index date was assigned according to the same hospital day as the matched exposed admission’s first qualifying exposure, to reduce immortal-time and exposure-opportunity bias.

Outcome definitions

The primary outcome was the development of AKI after the index date. AKI was defined and staged according to Kidney Disease: Improving Global Outcomes (KDIGO) criteria, using serum creatinine change and urine output when accurately documented. Serum creatinine-based AKI was the principal basis for outcome classification because creatinine data were consistently available across the cohort, whereas urine output was used only when reliable charted values were present.

AKI severity was classified according to the maximum AKI stage reached during hospitalization. Stage 2 or Stage 3 AKI was considered severe AKI. Stage 1 AKI was further described as Stage 1a and Stage 1b for clinical interpretation. For this study, Stage 1a AKI was defined as an absolute serum creatinine increase of ≥0.3 mg/dL without reaching a 1.5-fold increase from baseline. Stage 1b AKI was defined as a ≥1.5-fold increase from baseline but not meeting Stage 2 criteria. Stage 1a represented milder creatinine-defined AKI, whereas Stage 1b represented a more clinically meaningful Stage 1 event. A sensitivity analysis was therefore performed after excluding Stage 1a AKI from the endpoint definition, so that the conservative AKI outcome included Stage 1b, Stage 2, and Stage 3 events only.

Secondary outcomes included maximum AKI stage, Stages 2-3 AKI, time from index date to AKI, in-hospital mortality, hospital length of stay, vancomycin concentration patterns among admissions with available vancomycin-level data, and medication-specific AKI-overlapping exposure burden.

Data collection

Clinical and laboratory data were collected from inpatient medical records, medication administration records, laboratory reports, and radiology reports. Baseline variables included age, sex, body mass index, diabetes mellitus, congestive heart failure, baseline serum creatinine, estimated glomerular filtration rate, blood urea nitrogen, and intensive care unit status on the day of matching. The estimated glomerular filtration rate was calculated from baseline serum creatinine using standard clinical reporting methods.

Serum creatinine values were followed after the index date until discharge, death, or the last available inpatient renal function measurement. For patients who received vancomycin and had available therapeutic drug-monitoring data, the initial and maximum vancomycin concentrations were recorded. Markedly elevated initial vancomycin concentrations were additionally categorized using thresholds of greater than 30 µg/mL and greater than 40 µg/mL.

Medication exposure-burden analysis

Medication-specific nephrotoxic burden was assessed using exposure patient days. A patient-day was considered exposed if the patient received the relevant nephrotoxic medication or remained within the defined exposure window for iodinated contrast. AKI-associated exposure days were calculated when medication exposure overlapped with AKI during hospitalization. These analyses were performed at both medication-class and individual-medication levels.

Medication classes were not treated as mutually exclusive because a single admission could contribute exposure days to more than one nephrotoxic class. Therefore, medication-specific exposure-day analyses were considered descriptive and were not interpreted as direct comparative causal estimates of nephrotoxic risk.

Propensity-score matching

Propensity-score matching was used to reduce baseline differences between admissions with high nephrotoxic exposure and admissions without high exposure. The propensity score represented the probability of receiving high nephrotoxic exposure based on measured baseline clinical variables. Matching variables included age, sex, body mass index, diabetes mellitus, congestive heart failure, baseline serum creatinine, estimated glomerular filtration rate, blood urea nitrogen, and intensive care unit status on the day of matching.

The propensity score was estimated using logistic regression, with high nephrotoxic exposure status as the dependent variable. Covariates included age, sex, body mass index, diabetes mellitus, congestive heart failure, baseline serum creatinine, estimated glomerular filtration rate, blood urea nitrogen, and intensive care unit status on the day of matching. Exposed admissions were matched 1:1 to unexposed control admissions using nearest-neighbor matching without replacement. No outcome variables were included in the propensity-score model. Post-matching balance was assessed using standardized mean differences, with values <0.10 considered acceptable.

Statistical analysis

Continuous variables were summarized as mean with standard deviation or median with interquartile range, depending on distribution. Categorical variables were expressed as frequency and percentage. Between-group comparisons were performed using the independent-samples t-test or Mann-Whitney U test for continuous variables and the chi-square test or Fisher’s exact test for categorical variables, as appropriate.

Binary outcomes were summarized using odds ratios with 95% confidence intervals. Crude marginal odds ratios were calculated from the displayed 2 × 2 outcome counts in the matched cohort. Time from index date to AKI was summarized descriptively among admissions that developed AKI. This analysis did not include admissions without AKI and, therefore, was not interpreted as a full time-to-event comparison. Time from index date to AKI was summarized descriptively among admissions that developed AKI. This analysis did not include admissions without AKI and, therefore, was not interpreted as a full time-to-event comparison. Hospital length of stay was compared between groups, but this result was interpreted cautiously because longer hospitalization may increase both the opportunity for nephrotoxic exposure and the probability of AKI detection.

A sensitivity analysis was conducted by excluding Stage 1a AKI from the AKI endpoint. This analysis was performed to determine whether the observed association between high nephrotoxic exposure and AKI remained present after removing milder, potentially transient creatinine changes. Vancomycin-level analyses were restricted to admissions with available vancomycin concentration data. Binary comparisons involving markedly elevated vancomycin thresholds were assessed using Fisher’s exact test because of small event counts. All statistical tests were two-sided. A p-value of less than 0.05 was considered statistically significant. Analyses were conducted using IBM SPSS Statistics version 29.0 statistical software (IBM Corp., Armonk, NY).

## Results

A total of 1,500 adult inpatient admissions were included in the pre-matching cohort. Among these, 240 admissions had high nephrotoxic medication exposure, representing 16.0% of the study population. After propensity-score matching, all 240 exposed admissions were matched with 240 unexposed control admissions.

After matching, the exposed and control groups were generally comparable across most measured baseline characteristics. Age, sex distribution, body mass index, diabetes mellitus, congestive heart failure, baseline serum creatinine, estimated glomerular filtration rate, and intensive care unit status showed acceptable post-matching balance. However, blood urea nitrogen remained imbalanced, with a standardized mean difference of 0.211. This residual imbalance may reflect differences in renal perfusion, hydration status, catabolic burden, or illness severity and should temper causal interpretation of AKI outcomes (Table 1).

**Table 1:** Baseline characteristics of the propensity-score matched cohort Values are presented as median (interquartile range) or number (%), as appropriate. Standardized mean differences were used to assess post-matching covariate balance, with values <0.10 considered acceptable. Blood urea nitrogen showed residual imbalance after propensity-score matching. eGFR: estimated glomerular filtration rate; ICU: intensive care unit; IQR: interquartile range; SMD: standardized mean difference

Characteristic	Exposed group (n = 240)	Control group (n = 240)	Standardized mean difference
Age, years, median (IQR)	60 (48-69)	59 (44-69)	0.083
Female sex, n (%)	110 (45.8)	115 (47.9)	0.044
Body mass index, median (IQR)	29 (24-35)	29 (25-34)	0.060
Diabetes mellitus, n (%)	36 (15.0)	33 (13.8)	0.037
Congestive heart failure, n (%)	23 (9.6)	20 (8.3)	0.049
Baseline creatinine, mg/dL, median (IQR)	0.7 (0.6-0.9)	0.7 (0.6-0.8)	0.064
Baseline eGFR, mL/min/1.73 m², median (IQR)	102 (91-113)	104 (92-115)	0.088
Blood urea nitrogen, mg/dL, median (IQR)	14 (10-19)	13 (9-19)	0.211
ICU on day of match, n (%)	8 (3.3)	5 (2.1)	0.088

In the matched cohort, AKI occurred more frequently among admissions with high nephrotoxic medication exposure than among matched controls. AKI developed in 61 exposed admissions and 38 control admissions, corresponding to 25.4% and 15.8%, respectively. This yielded a crude odds ratio of 1.81. Stages 2-3 AKI were also numerically more frequent in the exposed group, although this comparison did not reach statistical significance. In-hospital mortality was numerically higher among exposed admissions, but the event count was small, and the difference was not statistically significant. Length of stay was longer in the exposed group; however, this finding should be interpreted cautiously because longer hospitalization may increase both exposure opportunity and AKI ascertainment (Table 2).

**Table 2:** Clinical outcomes in the propensity-score matched cohort Values are presented as median (interquartile range) or number (%), as appropriate. ORs and 95% CIs are presented for binary outcomes only and are crude marginal estimates derived from the displayed 2 × 2 counts. *Statistically significant at p < 0.05. AKI: acute kidney injury; CI: confidence interval; IQR: interquartile range; NA: not applicable; OR: odds ratio

Outcome	Exposed group (n = 240)	Control group (n = 240)	OR	95% CI	p-value
AKI, n (%)	61 (25.4)	38 (15.8)	1.81	1.15-2.85	0.013*
Stages 2-3 AKI, n (%)	15 (6.3)	7 (2.9)	2.22	0.89-5.54	0.125
Time from match to AKI among AKI cases, days, median (IQR)	3 (2-6)	2 (1-4)	NA	NA	<0.001*
In-hospital mortality, n (%)	6 (2.5)	3 (1.3)	2.03	0.50-8.20	0.504
Length of stay, days, median (IQR)	7 (4-11)	5 (4-9)	NA	NA	<0.001*

Among admissions that developed AKI, most events were mild. Stage 1b was the most frequent maximum AKI stage in both groups. Stage 3 AKI occurred in six exposed admissions and two control admissions, suggesting a possible shift toward more severe renal injury among exposed admissions. However, the small number of severe AKI events limits precision and prevents strong inference regarding AKI severity progression (Table 3).

**Table 3:** Maximum AKI stage among admissions who developed AKI Percentages were calculated among admissions that developed acute kidney injury within each exposure group. Stage-specific counts sum to the total number of AKI events in each group. AKI: acute kidney injury

AKI stage	Exposed group with AKI (n = 61)	Control group with AKI (n = 38)
Stage 1a, n (%)	19 (31.1)	14 (36.8)
Stage 1b, n (%)	27 (44.3)	17 (44.7)
Stage 2, n (%)	9 (14.8)	5 (13.2)
Stage 3, n (%)	6 (9.8)	2 (5.3)

A sensitivity analysis was performed after excluding Stage 1a AKI from the endpoint definition. Under this more conservative definition, clinically meaningful AKI, defined as Stage 1b or higher, remained more frequent in the exposed group. AKI excluding Stage 1a occurred in 42 exposed admissions and 24 control admissions, corresponding to 17.5% and 10.0%, respectively. This finding supports the robustness of the primary AKI association, although individual Stage 2 and Stage 3 outcomes remained underpowered (Table 4).

**Table 4:** Sensitivity analysis after excluding Stage 1a AKI Stage 1a AKI was excluded from the sensitivity endpoint. AKI excluding Stage 1a includes Stage 1b, Stage 2, and Stage 3 events only. ORs and 95% CIs are presented for binary outcomes only and are crude marginal estimates derived from the displayed 2 × 2 counts. *Statistically significant at p < 0.05. AKI: acute kidney injury; CI: confidence interval; IQR: interquartile range; NA, not applicable; OR: odds ratio

Outcome	Exposed group (n = 240)	Control group (n = 240)	OR	95% CI	p-value
AKI excluding Stage 1a, n (%)	42 (17.5)	24 (10.0)	1.91	1.12-3.27	0.024*
Stage 2 AKI, n (%)	9 (3.8)	5 (2.1)	1.83	0.60-5.55	0.417
Stage 3 AKI, n (%)	6 (2.5)	2 (0.8)	3.05	0.61-15.27	0.285
Time from match to AKI among AKI cases, days, median (IQR)	4 (2-7)	3 (1-6)	NA	NA	0.010*

Among admissions with available vancomycin-level data, patients who developed AKI had slightly higher initial vancomycin concentrations and higher maximum vancomycin concentrations than those who did not develop AKI. The difference was more pronounced for the maximum vancomycin concentration. However, the maximum vancomycin level may occur after AKI onset and may therefore reflect reduced renal clearance rather than preceding nephrotoxic exposure. Markedly elevated initial vancomycin concentrations above 30 µg/mL or 40 µg/mL were uncommon and did not differ significantly by AKI status. Vancomycin-level analysis was restricted to admissions with available vancomycin therapeutic drug-monitoring data. This subgroup included admissions from the matched cohort with documented vancomycin concentrations and was analyzed according to AKI status (Table 5).

**Table 5:** Vancomycin concentrations according to AKI status among admissions with available vancomycin-level data Values are presented as median (interquartile range) or number (%), as appropriate. Binary p-values were recalculated from the displayed counts using Fisher’s exact test. *Statistically significant at p < 0.05. AKI: acute kidney injury; IQR: interquartile range

Characteristic	No AKI (n = 186)	Developed AKI (n = 54)	p-value
Initial vancomycin level, µg/mL, median (IQR)	12 (9-16)	13 (10-18)	0.047*
Maximum vancomycin level, µg/mL, median (IQR)	16 (11-21)	18 (14-23)	<0.001*
Initial vancomycin level >30 µg/mL, n (%)	4 (2.2)	2 (3.7)	0.616
Initial vancomycin level >40 µg/mL, n (%)	1 (0.5)	0 (0.0)	1.000

At the medication-class level, vancomycin accounted for the largest absolute number of AKI-associated exposure days, followed by iodinated contrast and piperacillin-tazobactam. Antivirals and calcineurin/mTOR inhibitors also showed substantial AKI-associated exposure burden. When interpreted as a proportion of total exposure patient days, calcineurin/mTOR inhibitors and antivirals showed relatively high AKI-associated exposure percentages. These findings should be interpreted descriptively because medication classes were not mutually exclusive, and patients could contribute exposure days to more than one medication class (Table 6).

**Table 6:** Major medication classes associated with AKI-overlapping exposure days These data describe exposure burden and do not establish medication-specific causal risk. ACEI: angiotensin-converting enzyme inhibitor; AKI: acute kidney injury; ARB: angiotensin receptor blocker; mTOR: mammalian target of rapamycin; NSAIDs: nonsteroidal anti-inflammatory drugs

Medication class	Total exposure patient days	Total AKI-associated exposure days	AKI-associated exposure days, %	Stage 1 AKI days	Stage 2 AKI days	Stage 3 AKI days
Vancomycin	856	255	29.8	136	79	40
Iodinated contrast	716	222	31.0	123	66	33
Piperacillin-tazobactam	443	160	36.1	80	51	29
Antivirals	394	198	50.3	100	64	34
ACEI/ARB	316	83	26.3	43	30	10
NSAIDs	203	32	15.8	21	5	6
Calcineurin/mTOR inhibitors	167	90	53.9	49	27	14

At the individual medication level, vancomycin accounted for the largest number of AKI-associated exposure days, followed by iopamidol and piperacillin-tazobactam. Acyclovir and tacrolimus also showed notable AKI-associated exposure burden, consistent with the class-level findings for antivirals and calcineurin/mTOR inhibitors. However, absolute AKI-associated exposure-day counts are influenced by how frequently each medication was used. Therefore, these findings should be viewed as descriptive exposure-burden data rather than direct comparative estimates of nephrotoxic risk (Table 7).

**Table 7:** Top individual medications by AKI-associated exposure days Total AKI-associated exposure days equal the sum of Stage 1, Stage 2, and Stage 3 AKI days. AKI-associated exposure percentage was calculated as total AKI-associated exposure days divided by total exposure patient days. AKI: acute kidney injury

Medication	Total exposure patient days	Total AKI-associated exposure days	AKI-associated exposure days, %	Stage 1 AKI days	Stage 2 AKI days	Stage 3 AKI days
Vancomycin	856	255	29.8	136	79	40
Iopamidol	603	183	30.3	104	54	25
Piperacillin-tazobactam	443	160	36.1	80	51	29
Acyclovir	219	108	49.3	52	36	20
Tacrolimus	155	86	55.5	46	26	14
Lisinopril	189	52	27.5	30	16	6
Valacyclovir	104	50	48.1	31	13	6
Losartan	102	24	23.5	12	9	3
Iodixanol	38	20	52.6	9	6	5
Valganciclovir	33	18	54.5	11	5	2

## Discussion

In this study, high nephrotoxic medication exposure and/or iodinated contrast exposure were associated with a higher frequency of in-hospital AKI among adult inpatients. AKI occurred in 25.4% of exposed admissions compared with 15.8% of matched controls, corresponding to nearly 1.8-fold higher odds of AKI. This association remained present even after excluding Stage 1a AKI, where clinically more meaningful AKI continued to be higher in exposed patients. This is an important internal validation of the finding, because it shows that the observed association was not driven only by small or transient serum creatinine changes. Most AKI episodes were mild; however, Stage 3 AKI was numerically more frequent in exposed patients. Exposed admissions also had longer hospital stays and numerically higher mortality, although these latter outcomes were limited by small event counts. Medication-burden analysis further showed that vancomycin, iodinated contrast, piperacillin-tazobactam, antivirals, and calcineurin/mTOR inhibitors contributed substantially to AKI-overlapping exposure days. At the individual-drug level, vancomycin, iopamidol, piperacillin-tazobactam, acyclovir, tacrolimus, lisinopril, valacyclovir, iodixanol, and valganciclovir emerged as clinically relevant exposure signals. Therefore, the present study does not merely show that AKI was common; it identifies a modifiable inpatient risk environment where drug burden, contrast exposure, renal monitoring, and patient susceptibility intersect.

The present findings closely agree with the adult NINJA-based study by Griffin et al. [12], in which high nephrotoxic exposure was present in 16% of adult admissions, and AKI was higher among exposed patients than matched controls, 26% versus 16%, with an odds ratio of 1.82. The similarity is striking because the present study also found 16.0% high exposure in the pre-matched cohort and an AKI odds estimate of almost the same magnitude. Both studies also converged on the same practical “usual suspects”: vancomycin, iodinated contrast, and piperacillin-tazobactam. This consistency strengthens the argument that nephrotoxin surveillance should not remain confined to pediatric practice or intensive care settings but should be adapted to routine adult inpatient care.

The findings are also supported by Garcia et al. [6], who reported that drug-induced AKI accounted for 19.3% of hospitalized AKI cases, with vancomycin and contrast agents as the leading nephrotoxins. Importantly, Garcia et al. [6] showed that most drug-induced AKI was not isolated; nearly three-fourths occurred with ischemia and/or sepsis. This observation fits the biological reading of the present cohort. Nephrotoxic exposure is rarely a pure toxic insult acting in isolation. It usually operates over a background of infection, dehydration, haemodynamic stress, catabolism, reduced renal reserve, and therapeutic urgency.

Mechanistically, the results are coherent with Perazella [7] and Perazella and Rosner [8], who conceptualized drug-induced AKI as a layered interaction between drug factors, patient vulnerability, and kidney-specific handling of toxins. Vancomycin and aminoglycosides may cause proximal tubular injury through intracellular accumulation and oxidative or mitochondrial stress; beta-lactams and nonsteroidal anti-inflammatory drugs may contribute through acute interstitial nephritis; antivirals, such as acyclovir, may produce crystal-associated tubular obstruction; and calcineurin inhibitors may induce renal vasoconstriction and tubular injury. Thus, the medication-burden pattern in the present study is pathophysiologically credible.

The antibiotic signal is consistent with Clifford et al. [4], who identified strong AKI signals for polymyxins, aminoglycosides, vancomycin, trimethoprim-sulfamethoxazole, and penicillin combinations, and emphasized prevention through risk assessment, dose adjustment, therapeutic monitoring, de-escalation, and correction of hypovolemia. Similarly, Khalili et al. [5] reported antibiotic-associated AKI in 17.9% of infectious-disease ward patients, with aminoglycosides, amphotericin B, vancomycin, and beta-lactam combinations repeatedly implicated. Their observation that diabetes, dehydration, and concomitant nephrotoxins independently increased AKI risk is clinically relevant to the present cohort, where residual blood urea nitrogen imbalance may partly reflect subtle volume or illness-severity differences.

The predominance of mild AKI in the present study should not be interpreted as trivial. Cheng et al. [9] showed that AKI was frequently missed in hospitalized adults, particularly when the injury was mild or clinically masked. Esposito et al. [10] similarly demonstrated that undetected biochemical AKI was common and independently associated with mortality. Zhang et al. [11], in diuretic-associated AKI, also found a striking underdiagnosis rate, reinforcing that routine creatinine-defined AKI is often clinically under-recognized. In this context, the persistence of the association after excluding Stage 1a AKI is meaningful. It suggests that the nephrotoxin signal survived a more conservative clinical definition and was not simply a laboratory artefact.

The present findings also resonate with broader AKI prevention literature. Li et al. [1] framed AKI as a global health threat that is common, harmful, and often preventable. Yamout et al. [3] found that nearly one-third of hospital-acquired AKI episodes were potentially preventable, commonly through contrast prophylaxis, correction of hypotension or volume depletion, and avoidance of multiple nephrotoxic insults.

Unlike Griffin et al. [12], where high nephrotoxin exposure was significantly associated with Stages 2-3 AKI and mortality, the present study found only numerical increases in severe AKI and in-hospital death. This difference should be interpreted cautiously rather than as a contradiction. The present matched cohort was smaller, with only 480 admissions after matching, and severe events were infrequent. The intensive care unit proportion was also low after matching, which likely reduced the absolute number of deaths and severe AKI episodes. In addition, patients with pre-existing AKI were excluded, and propensity matching reduced measurable baseline-risk differences between groups. These design choices strengthened internal comparison but may have attenuated differences in downstream outcomes.

The vancomycin findings also require careful interpretation. Patients who developed AKI had higher initial and maximum vancomycin concentrations, but markedly elevated initial levels above 30 or 40 µg/mL were uncommon and not statistically different. This pattern is biologically plausible. Initial exposure may contribute to injury in susceptible patients, but maximum vancomycin levels may also rise after AKI develops because impaired renal clearance reduces drug elimination. Therefore, the maximum vancomycin level may be both a cause and a consequence of AKI. Without area under the concentration-time curve (AUC)-guided dosing data and exact temporal alignment between vancomycin levels and creatinine rise, causality cannot be firmly assigned.

The relatively mild AKI profile may also reflect earlier detection, exclusion of admission AKI, and use of serum creatinine surveillance in a prospective design. Studies enriched for intensive care patients, sepsis, cirrhosis, intracerebral hemorrhage, or COVID-19 understandably report higher Stages 2-3 AKI and mortality because their patients enter the risk window with heavier systemic inflammation, organ failure, and hemodynamic instability. By contrast, the present cohort captures a broader ward-and-ICU inpatient population, where nephrotoxic injury may appear first as mild biochemical AKI. That does not make it benign. It makes it actionable.

This study has several strengths. First, the prospective observational design allowed systematic capture of nephrotoxic exposure and subsequent renal outcomes in real-world adult inpatient care. Second, propensity-score matching improved comparability between exposed and unexposed admissions across age, sex, body mass index, diabetes, congestive heart failure, baseline creatinine, estimated glomerular filtration rate, blood urea nitrogen, and intensive care unit status. Third, exclusion of patients with AKI before nephrotoxic exposure reduced reverse classification of pre-existing renal injury as exposure-associated AKI. Fourth, the sensitivity analysis excluding Stage 1a AKI strengthened the clinical interpretation by showing that the association persisted beyond very mild creatinine-defined events. Fifth, the medication-class and individual-drug exposure-day analysis adds practical value. It moves the discussion from a generic statement of “nephrotoxic risk” to a bedside surveillance list: vancomycin, contrast agents, piperacillin-tazobactam, acyclovir/valacyclovir/valganciclovir, and tacrolimus. This is useful for clinicians, pharmacists, and nephrologists because it identifies where monitoring effort should be concentrated.

The findings should still be interpreted with caution. Propensity matching reduced measured confounding, but it cannot remove unmeasured differences in sepsis severity, hemodynamic instability, fluid balance, nutrition, inflammatory burden, antimicrobial indication, contrast indication, or clinician decision-making. Residual blood urea nitrogen imbalance may reflect subtle renal hypoperfusion, dehydration, catabolic state, or illness severity. Urine-output data were not uniformly reliable, so AKI classification depended mainly on serum creatinine. This may underestimate early or oliguric AKI. Medication classes were overlapping and not mutually exclusive, making exposure-day analysis descriptive rather than causal. Longer hospital stay may be both a consequence of AKI and an opportunity for more nephrotoxic exposure and more creatinine testing. Vancomycin-level analysis was limited by incomplete temporal information, the absence of AUC-guided exposure metrics, and possible reverse causality. Severe AKI, dialysis, and mortality analyses were underpowered because event counts were small. Finally, long-term renal recovery, chronic kidney disease progression, and post-discharge mortality were not assessed.

## Conclusions

This prospective propensity-score-matched cohort demonstrates that high nephrotoxic medication exposure and/or iodinated contrast use are associated with a significantly higher frequency of in-hospital AKI among adult inpatients. The persistence of this association after excluding very mild Stage 1a events indicates that the renal injury signal was clinically meaningful, not merely a transient biochemical fluctuation. Although severe AKI and mortality were only numerically increased, the pattern suggests that nephrotoxic burden may still amplify early renal vulnerability, especially when combined with infection, hemodynamic stress, dehydration, diabetes, heart failure, or rising blood urea nitrogen. Vancomycin, iodinated contrast, piperacillin-tazobactam, antivirals, and calcineurin/mTOR inhibitors emerged as key exposure targets for surveillance. These findings support routine nephrotoxin stewardship, baseline renal-risk assessment, estimated glomerular filtration rate (eGFR)-based dose adjustment, hydration optimization, avoidance of unnecessary combinations, and scheduled creatinine monitoring. Early multidisciplinary review may reduce preventable AKI in hospitalized adults while preserving necessary antimicrobial and diagnostic care in real-world practice.

## References

[1] Li PK, Burdmann EA, Mehta RL (2013). Acute kidney injury: global health alert. Kidney Int.

[2] Yokota LG, Sampaio BM, Rocha EP, Balbi AL, Sousa Prado IR, Ponce D (2018). Acute kidney injury in elderly patients: narrative review on incidence, risk factors, and mortality. Int J Nephrol Renovasc Dis.

[3] Yamout H, Levin ML, Rosa RM, Myrie K, Westergaard S (2015). Physician prevention of acute kidney injury. Am J Med.

[4] Clifford KM, Selby AR, Reveles KR, Teng C, Hall RG 2nd, McCarrell J, Alvarez CA (2022). The risk and clinical implications of antibiotic-associated acute kidney injury: a review of the clinical data for agents with signals from the Food and Drug Administration's Adverse Event Reporting System (FAERS) database. Antibiotics (Basel).

[5] Khalili H, Bairami S, Kargar M (2013). Antibiotics induced acute kidney injury: incidence, risk factors, onset time and outcome. Acta Med Iran.

[6] Garcia G, Pacchini VR, Zamoner W, Balbi AL, Ponce D (2024). Drug-induced acute kidney injury: a cohort study on incidence, identification of pathophysiological mechanisms, and prognostic factors. Front Med (Lausanne).

[7] Perazella MA (2018). Pharmacology behind common drug nephrotoxicities. Clin J Am Soc Nephrol.

[8] Perazella MA, Rosner MH (2022). Drug-induced acute kidney injury. Clin J Am Soc Nephrol.

[9] Cheng X, Wu B, Liu Y, Mao H, Xing C (2017). Incidence and diagnosis of Acute kidney injury in hospitalized adult patients: a retrospective observational study in a tertiary teaching Hospital in Southeast China. BMC Nephrol.

[10] Esposito P, Cappadona F, Marengo M (2024). Recognition patterns of acute kidney injury in hospitalized patients. Clin Kidney J.

[11] Zhang R, Liu Y, Cao J (2022). The incidence and risk factors analysis of acute kidney injury in hospitalized patients received diuretics: a single-center retrospective study. Front Pharmacol.

[12] Griffin BR, Wendt L, Vaughan-Sarrazin M (2023). Nephrotoxin exposure and acute kidney injury in adults. Clin J Am Soc Nephrol.

